# Educational Activities for Students and Citizens Supporting the One-Health Approach on Antimicrobial Resistance

**DOI:** 10.3390/antibiotics10121519

**Published:** 2021-12-11

**Authors:** Massimiliano Marvasi, Lilliam Casillas, Alberto Vassallo, Diane Purchase

**Affiliations:** 1 Department of Biology, University of Florence, 50019 Sesto Fiorentino, Italy; massimiliano.marvasi@unifi.it (M.M.); alberto.vassallo@unifi.it (A.V.); 2 Biology Department, University of Puerto Rico-Humacao, Humacao, PR 00792, USA; lilliam.casillas@upr.edu; 3 Department of Natural Sciences, Middlesex University, London NW4 4BT, UK

**Keywords:** One-Health, antimicrobial resistances, antibiotics, informal education, formal education

## Abstract

Antibiotic resistance is one of the biggest threats to global health, food security and development. Urgent action is needed at all levels of society to reduce the impact and spread of antibiotic resistance. For a more sustaining approach, education in children, college students, citizens and caregivers are essential. The One-Heath approach is a collaborative, multisectoral and transdisciplinary strategy in which, no single organizations or sector can address the issue of antimicrobial resistance at the human–environment interface alone. Within this strategy, education plays a central role. In this scoping review, we highlighted a range of learning activities on antibiotic resistance as part of the One-Health approach. In particular, those applications that can be introduced to a wide audience to help arrest the current crisis for the next generation. The review identifies a high number of teaching opportunities: board and role-play games, round tables, musicals, e-learning and environmental experiments to couple with more curricula and formal education to inform a diverse group of audiences.

## 1. Introduction

Infections due to antibiotic-resistant bacteria threaten modern healthcare and the irrational use (by both professionals and final users) of important antibiotics has been reported [1,2]. Such activities lead to an increased risk of spreading antimicrobial resistance in microbial populations. Moreover, the so-called ‘antimicrobials of last resort’, such as vancomycin, are becoming less effective to treat infectious diseases [2,3]. In this context, antimicrobial resistance must be considered as a medical emergency. 

Education has an important role in arresting the serge of antimicrobial resistance and mitigating the risk of a post-antibiotic era. In 2018, the UK Heath Education England Department, National Health Service (HEE-NHS), released a report on educational priorities to tackle antimicrobial resistance based on feedback from more than 1000 health professionals and healthcare educators in England. Among the key themes, the HEE-NHS promoted education at the following two levels: education of healthcare students, and community education (including school students, society and non-healthcare professional students) [4]. Two recent surveys advocate a UK national competency standard for healthcare students in training in the use of antibiotics [5,6]. These competencies were grouped into domains that accounted for the prevention and control of infections, antibiotics and antibiotic resistance, infection diagnosis and antibiotic use, antibiotic prescription practices, person-centered care and the promotion of collaborative practice among various professionals [5,6]. Undergraduate education is the main route to prepare healthcare professionals on antimicrobial stewardship and should be strengthened via continued education programs.

With reference to the role of education on antibiotic resistances in low-income, middle-income countries (LMIC) and high-income countries (HIC), the disparities are reflected in the differences in the rise of resistances. It is often assumed that LMIC have less access to infrastructure and high-quality education for all the citizens, which accounts for the high increase in antibiotic resistance. However, counterintuitively, studies show that a high GDP per capita and high education are associated with the high development of resistances because higher education is correlated with high antibiotic consumption (high GDP per person has high antibiotic usage) [7,8,9]. This indicates AMR education rather than the GDP per capita has a stronger influence in the spread of antibiotic resistance. Nevertheless, in LMIC, antibiotic use has increased substantially (over the same period measured in HIC) as a result of economic improvements and changes in diet. Therefore, the role of education needs to be shaped and strengthened to better inform citizens. The One-Health approach addresses the limitation of generic formal education by promoting more pedagogical-targeted approaches to tackle the rise of antibiotic resistance. This scoping review proposes some of them (Box 1).

Box 1 **Definitions**

**Antimicrobial** is a wide term used to identify a compound able to selectively kill microorganisms. Bacteria, fungi or parasites are all microorganisms.
The term **antibiotics** is widely accepted for antimicrobials that specifically target bacteria.
**Antimicrobial resistance** and **antibiotic resistance** are terms that refer to the development of resistance to specific antibiotics or antimicrobials. Microorganisms find ways to survive. This can occur by restricting the access of the antimicrobial into the cell, degradation, and the complexation of the antibiotic. DNA mutations affecting the antibiotic target site is another way to acquire resistance.
The **One-Health** approach is a comprehensive process, which includes multiple disciplines (medicine, biology, social science, education, citizen science) that communicate and work together to achieve better public health outcomes [10,11]. In other words, the One-Heath approach is a collaborative, multisectoral and transdisciplinary strategy in which no single organization or sector can address the issue of antimicrobial resistance at the human–environment interface alone.
**Antimicrobial stewardship** involves interdisciplinary professionals in an effort to effect prudent antimicrobial use for patients [12]. For example, medical students should be better informed on the mechanisms of microbial evolution to foster an appreciation and awareness of antibiotic resistant development as a result of unnecessary antibiotic prescriptions [13]. Every medical student, during their professional practice, should aim to resolve the patient’s infection while minimizing the development of antimicrobial resistance [14].

Education has an important role within antimicrobial stewardship and the One-Health approach. It is clear that education for all type of pupils and students would ultimately lead to either well-informed adults that can appreciate the risk of increased antibiotic resistance and raise concerns about the over-prescription of antibiotics by medical practitioners, or health professionals to limit such practices [14,15].

**Figure 1:**
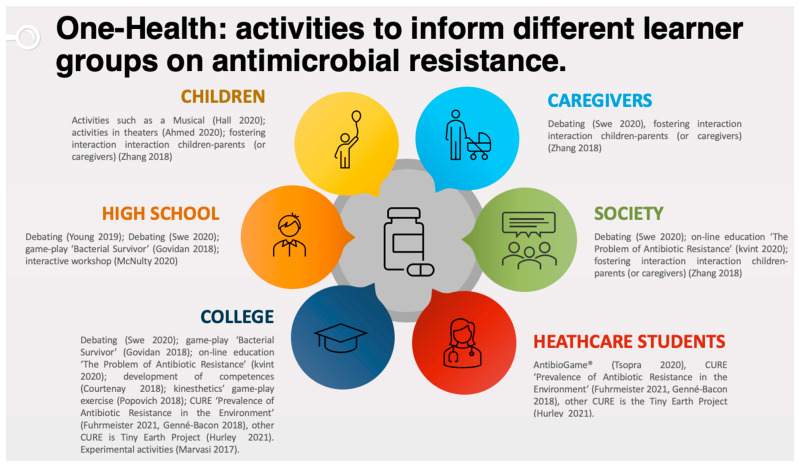
Educational activities for different learning groups on antimicrobial resistance. The picture shows the interconnection of six domains in which the antimicrobial One-Health approach should be integrated. CURE: Course-Based Undergraduate Research Experience. The graphic is adapted from the free resource, PresentationGO.com accessed on 8 September 2021.

Here, we highlight the current knowledge and efforts carried out to educate diverse populations on antimicrobial resistances. We propose a scoping review on selected papers that, within the One-Health approach, can be used as examples of pedagogical practices. Tackling this aspect early in schools is important and would lead to better informed citizens of tomorrow. These interconnections of teaching strategies/practices/interventions offer a better strategy in the form of the One-Health approach to educate practitioners and citizens (Figure 1).

In this review, we have gathered information on interactive learning and informal educational strategies that have been developed specifically in the context of educating students and citizens. This scoping review has taken into consideration the vast body of knowledge, its complexity and its heterogeneous nature to provide ‘good practice’ examples for educators to adapt and build on according to the specific learning environment and types of audiences.

## 2. Fewer Antibiotics for Youth and Adults (Non-Medical Students)

As previously stated, in the One-health approach, a number of activities have been proposed both at the informal and formal education level, such as in schools for students, teachers and the general public (Figure 1). These activities were able to foster students’ and citizens’ engagement by disseminating important public health messages. In many cases, the proposed activities do not only target a specific group (e.g., students) but learner groups are interchangeable and diverse: the audience can be composed, for example, of adults and/or secondary school students from different backgrounds. The different approaches and their merits and demerits are summarized in Table 1

### 2.1. Informal Education: Theater Presentations

Examples are available in primary schools, providing a positive holistic learning experience for children. One of them is the exposure of pupils from primary school (aged 9 to 11 years) to a musical for children entitled, ‘The Mould that Changed the World’ [16]. The musical is about the discovery of antibiotics, the risks of drug-resistant infections and the importance of prudent antibiotic use with the main aim being to educate and engage the public regarding the importance of conserving antibiotics and the concept of antimicrobial resistances. The intervention was implemented in two UK primary schools by drama and music specialists through a series of workshops, associated learning resources and by acting and music performances. Knowledge and long-term knowledge gain was demonstrated by motivating the participants to minimize personal antibiotic use and influence attitudes to antibiotics in their family and friends [16]. Forum theatre approaches have also been recently proposed by two different authors to convey the importance of microbial resistances. In both cases, the activities were structured as informal education to increase public awareness of antimicrobial resistance as a key component of effective antimicrobial One-Health strategies. Educational theatre combined with an expert panel was used to engage the public in deeper discussions on the bacterial acquisition of antibiotics resistance in a play entitled, ‘The drugs don’t work’ [17].

In another activity, simple but effective key messages based on the WHO’s world antibiotic awareness campaign were proposed, including the following: (i) antibiotics are medicines used to treat bacterial infections, (ii) they are not useful for coughs and colds, (iii) avoid use of leftover antibiotics, (iv) prevent infections by regularly washing hands, preparing food hygienically, avoiding close contact with sick people and keeping vaccinations up to date. The study reached 1175 community members and the forums had panels of experts [18]. The activity showed positive engagement with the public and students’ families and more importantly provided the knowledge needed.

### 2.2. Formal Education: Presential or Virtual Workshops

Within the One-Health framework, the children–parents (or caregivers) interactions present unique opportunities to strengthen learning about antimicrobial resistance. For example, as children’s diseases are frequently treated with antibiotics, educational interventions on the rationale for using specific antibiotics by caregivers have been proposed [19]. The authors suggested a pedagogical approach where the caregivers and children are both trained simultaneously, by using specific easy-to-read interactive training sessions, booklets and printed or electronic educational materials (e.g., from the US Center for Disease Control (CDC)). The materials could be brought home and studied (or interacted with) by the caregiver and the children [19]. Zhang et al. suggested the following four key main aspects to be focused on: knowledge on infectious agents or microorganisms, their routes to spread infection, the prevention of an infection and the rational use of antibiotics for respiratory diseases caused by bacteria but not viruses. After the period of learning, questionnaires were used to assess their understanding of each aspect. Specifically, Knowledge-Attitude-Practice (KAP) questionnaires were suggested to assess caregivers’ knowledge, practice and their attitude, while Knowledge-Retention (KR) questionnaires were suggested to assess children’s knowledge (notions of microbes, antibiotics and hygiene) [19].

Another activity proposed by Marvasi and colleagues [20] applied ecological concepts on the fitness of antibiotic-resistant and sensitive waterborne bacteria in undergraduate settings. In this activity, students learned how to set up a fitness experiment by using an isogenic pair of antibiotic-resistant and sensitive bacteria in the presence or absence of selective pressure. The students showed proficiency in analyzing the complexity of fitness data and understanding that the selective pressure in the environment can bring a selection of dangerous antibiotic resistant strains [20]. Furthermore, the activity, which involved water sampling from their communities, allowed the students to develop student citizen behaviors.

### 2.3. Combining Informal and Formal Education: Role Playing and Others

Learning through a ‘role-playing game’ approach is another interesting strategy to formally and informally introduce antimicrobial resistances and their principles [21]. A role-playing game is a game where participants assume the roles of characters and act consequently. Computers are not required in these activities, the approach fosters kinesthetics and tactile senses by an interactive role-playing exercise. The activity stimulates students by ‘acting’ as molecules, fosters a strong visualization of the molecular mechanisms (including the cell envelope and the peptidoglycan layer) and a clear understanding of the mechanism of action for β-lactam antibiotics, as well as the gene acquisition and protein conformational changes that can result in resistance. Interestingly, the kinesthetics’ exercise showed a long-term retention of the subject material in many students. A high level of student engagement was observed, and the activity received positive feedback [21]. Another in-class game activity called ‘Bacterial Survivor’ has been developed for a large audience and it can be completed in a large lecture setting [22]. Bacterial Survivor was a productive active learning strategy through a game modelling the random nature of genetic change. The game allowed only 15 min to combat specific misconceptions about antibiotic resistance in bacterial populations. This can be played by groups of hundreds of people, which makes it excellent for general Biology or Microbiology courses. Essentially, the activity allows for the clarification of the concept that mutations and horizontal gene transfers are random and that selective pressures is exerted by environmental changes [22].

The use of role-play in schools has proven to be an effective educational tool to teach antimicrobial resistances, especially when the topic relates to proper antibiotics’ prescription. The activity has been shown to develop different skills, including confidence and communication as well as gaining a greater understanding of the topic discussed [23]. More specifically, the activity uses the e-Bug antibiotic resistant debate kit, which is a structured practice debate on a controversial topic (https://e-bug.eu, accessed on 8 September 2021). Students act according with the character cards provided to them, one per group, and the students have a few minutes to read and prepare. Each card represents perspectives and points of view according to the different professional and cultural backgrounds of the proposed characters (e.g., different professionals). The debate is structured in ‘different rounds’, helping the students to think through the issues and reconsider their opinions [23]. Prescriptions debates have been delivered in seven schools across South West England to 235 students aged 13–16 years and the results show a significant improvement in knowledge for most of the areas covered, particularly regarding when to use antibiotics and bacteria developing resistance [23].

Other strategies have been used in peer-education projects; for example, university healthcare students taught other students in three UK schools (16–18 years old students) about self-care and antibiotic use for infections [24]. These teaching activities include interactive workshops and real-life illness scenarios and have been shown to increase confidence regarding the concepts of antimicrobial resistance in both groups (teachers and learners). Nevertheless, some disadvantages have been noted, such as stress associated with teaching peers and a reduced authoritative perception of the college teacher by the 16–18-year-old students [24].

### 2.4. Course-Based Research Experiences for Diverse and International AR Programs

Within formal education practices, the best examples of promoting antibiotic resistance awareness are the ‘course-based research experiences’ (CRE) or, specific for undergraduates, the ‘Course-Based Undergraduate Research Experience’ (CURE) [25]. These activities have been developed as a research-intense course specifically for bachelor or graduate programs. In some cases, such activities can cover an entire semester or be shortened to a few weeks within a given course. CUREs are known to foster active learning and are a driver for retention in all students. The American Society for Microbiology has identified CUREs as tools to broaden microbiology, introducing students to procedures to isolate bacteria from soil and urban water and characterize their antimicrobial sensitivity to both experimental antimicrobials and known pharmaceutical antibiotics [26]. Estrada et al. [27] have shown that students from underrepresented groups are more likely to persist in science when enrolled in CUREs. Moreover, the students’ sense of science efficacy and the identity of students from disadvantaged backgrounds improve, and students are motivated to do well in their research because the project was of interest to their communities [28]

While conducting CUREs, students learn authentic science practices within a broadly relevant project. An interesting activity has been proposed to educate students in this format, by studying the prevalence of antibiotic resistance in the environment using common but internationally established microbiological methods and equipment. The activity is called ‘Prevalence of Antibiotic Resistance in the Environment’ (PARE) and it has a double intention: combating antimicrobial resistance through student-driven research and fostering environmental global surveillance [25]. This activity (https://sites.tufts.edu/ctse/pare accessed on 8 September 2021) has engaged students around the globe, in the USA, France, Germany and China, in the surveillance of environmental soil samples to document the prevalence of antibiotic-resistant bacteria [25,29].

‘The Tiny Earth Project’ is a well-known international formal program for teaching antibiotic resistance (https://tinyearth.wisc.edu accessed on 8 September 2021). Currently, it hosts nearly 10,000 students around the world, across 45 US states and 15 countries [30]. Tiny Earth is a network of instructors and students focused on ‘student sourcing’ and ‘antibiotic discovery’ from the soil in their backyards. The mission of the program is to inspire students to pursue careers in science through original laboratory and field research conducted in introductory courses with the potential for a global impact. The program aims to address the worldwide health threat of having a diminishing supply of effective antibiotics to treat multi-resistant bacteria by tapping into the collective power of many student researchers concurrently to tackle the same challenge. In Tiny Earth, standard General Microbiology, General Biology and/or Cell and Molecular Biology courses are replaced or reinforced by the discovery-based experience [30].

### 2.5. Online Activities

The education of students and citizens can also be delivered online. A recent approach of online education has shown that distance learning can improve the awareness and understanding of antibiotic resistance for college students, teachers and the general public. An online multidisciplinary course has been proposed with a concise title ‘The Problem of Antibiotic Resistance’ and included videos on antibiotic resistance with topics ranging from chemistry to practical philosophy [31]. The course is available at https://antibiotic-resistance.org (accessed on 8 September 2021). The course received positive evaluations and students were examined based on important learning objectives, such as fostering an understanding of the issue, the reasons behind today’s crisis, explaining the origin of antibiotic resistances, how to reduce it and a description of research aimed at coping with the problem [31].

## 3. Activities for Medical Students

Several activities that promote learning among healthcare students have identified the knowledge gap in this student group. For example, a study designed to ascertain the knowledge of medical students from three main universities in the US showed that most students did not know the appropriate management of complicated urinary tract infections or antimicrobial-resistant infections, and only half successfully recognized the spectrum of activity of certain commonly used antimicrobials [32]. Furthermore, the perceptions and knowledge scores of the respondents suggested that there were deficiencies in the US medical school curricula, including fundamental concepts of antimicrobial usage. Interestingly, similar studies conducted in different countries such as China, Colombia, Singapore and Italy have shown similar results [33]. A formal evaluation of the curricula of medical schools regarding education about antimicrobials needs to be evaluated, and the introduction of concepts of the molecular aspects of bacterial resistance in general microbiology courses is strongly recommended. Recently, an informal teaching resource has been proposed to improve the training of medical students in antibiotic use in primary care: the AntibioGame^®^. This game, based on role-play, proposes clinical case templates built from a list of learning goals fostering the analysis of clinical practice. The scenarios used in the game are realistic and cover situations frequently encountered in primary care [34].

Pharmacy students are another important student population that should be an integral part of the AMR educational reform. According to a survey on knowledge of antimicrobials, pharmacy students showed significant variability in their preparation for antimicrobial stewardship and their knowledge was dependent on their previous school education. Due to their relevant role in antimicrobial stewardship, when selling antibiotics to the general population, better education for a pharmacist is of primary importance to the One-Health alliance [32]. Similar attention should be placed on veterinary students, since these professionals are involved in the issue when administering antibiotics. A recent study on about 3000 veterinary students showed gaps in knowledge and practices concerning antibiotic resistance. These gaps will likely contribute to inadequate use in their future practice and to the release of antibiotics into the environment [35].

## 4. Conclusions and Perspectives

In summary, we have highlighted the collaborative, multisectoral and transdisciplinary strategies that can be integrated into the One-Heath approach. Significant efforts have been made in the education sector to improve the effectiveness of the educational trajectories on antimicrobial resistances all over the world. This scoping review shows many activities, both formal and informal, to combat antibiotic microbial resistance. However, the continued education of healthcare professionals (stewardship programs for professional) is out of the scope of this review, since we focused on pre-professional stages or less informed learners. It is imperative to broaden microbiology concepts and make it accessible to all, transcending age, race and educational level. A One-Health approach must be implemented to revitalize medical schools’ curricula and standardize the competency of all healthcare professionals regarding the basis of microbial resistance [5,6]. The integration of discovery and research-based strategies should be implemented in high schools and undergraduate courses [36]. One critical route to influence and engage more people from diverse societies and social backgrounds is by fostering their inclusion in science, technology, engineering and mathematics (STEM). In the US, the number of people excluded due to their ethnicity and race (PEERs) in STEM fields is high [37]. According to the US National Science Foundation (NSF, Alexandria, VA, USA), only 18% of undergraduates and 7% of doctoral degrees or STEM faculty members are PEERs [38]. Studies from the National Center for Science and Engineering Statistics (National Science Foundation, 2016) have shown a positive trend where the number of PEERs entering college intending to study STEM has nearly tripled since 1992; however, only 50% of the entrants gain an undergraduate degree, with the majority leaving after their freshman year. This high attrition rate results in the loss of opportunities to raise awareness of antimicrobial resistance through higher education. In addition, the knowledge and appreciation of cultural differences should be taken into classrooms, by having people from different backgrounds conducting research on the antibiotic resistance field: approaching the same problem from different perspectives can raise awareness regarding cultural biases.

Informal and formal strategies for antimicrobial stewardship are diverse and have great content; however, they are limited to the awareness and attitudes of the clinical professionals that implement them. Citizen education on resistances to antibiotics is, therefore, vital to achieve a community impact, but the provisions are quite diffuse all over the world [39,40]; we recommend a central or regional repository to share best practice. Educators also need better resources to communicate effectively. One good example of e-learning is the e-Bug resource (www.e-bug.eu accessed on 8 September 2021) [41]. This website contains suitable tools to aid educator learning for continuous professional development by improving knowledge, confidence and skills. The research shows that awareness in the area of antimicrobial resistance is variable in students of health- (including medicine, pharmacy and veterinary science) and non-health-related majors [42]. Therefore, there is a pressing need to implement curricula and activities: this educational trajectory should start from pupils in secondary (or high) school, and extend to those studying for bachelor and higher degrees [14]. We acknowledge that the proposed educational activities are far from the ideal experiments (e.g., the proposed activities do not have equivalent teachers and learner groups, neither do pure control groups exist) and the levels of confidence in the interventions are limited, but the activities can be easily implemented to mitigate the threat of antimicrobial resistance via the One-Health approach.

## Figures and Tables

**Table 1:** Merits and the demerits of the type of learning proposed.

Type of Activity	Merits	Demerits
Informal education: Theater presentations	These activities are driven by learners’ interest and have been developed for a wide range of audiences and children.	As the target audiences are the general public and children, the activities tend to be at a superficial level of understanding.
Formal education: Presential or virtual workshops	These activities reach deeper explanation and concepts.	Level of participation is dependent on students’ interest and motivation. It may require studying some concepts in advance.
Combining informal and formal education	Useful to stimulate less interested students.	Students with prior knowledge may become disinterested and less engaged.
Course-based research experiences	The best in terms of understanding, motivation and interest.	Highly resources-intensive in terms of instructors’ involvement and consumables. It needs to be carefully organized.
Online activities	Activities always available. After preparation, they require relatively low maintenance from the instructor.	It attracts only motivated students and interested audiences.
